# Different *WDR36* mutation pattern in Chinese patients with primary open-angle glaucoma

**Published:** 2009-04-03

**Authors:** Bao Jian Fan, Dan Yi Wang, Ching-Yu Cheng, Wendy Charles Ko, Shun Chiu Lam, Chi Pui Pang

**Affiliations:** 1 Department of Ophthalmology & Visual Sciences, the Chinese University of Hong Kong, Hong Kong, China; 2 Department of Ophthalmology, National Yang Ming University School of Medicine and Taipei Veterans General Hospital, Taipei, Taiwan; 3 Department of Ophthalmology, Harvard Medical School, Massachusetts Eye and Ear Infirmary, Boston, MA

## Abstract

**Purpose:**

To determine the distribution of WD repeat domain 36 (*WDR36*) sequence variants in Chinese patients with primary open-angle glaucoma (POAG).

**Methods:**

One hundred and thirty-five unrelated POAG patients (82 high tension glaucoma [HTG], 42 normal tension glaucoma [NTG], and 11 juvenile-onset POAG [JOAG] patients) and 77 unrelated controls were recruited. All 23 coding exons and splicing junctions of *WDR36* were sequenced using BigDye^®^ Terminator v3.1 cycle sequencing kit. Single nucleotide polymorphism (SNP) and haplotype associations were analyzed using PLINK (version 1.04).

**Results:**

Nineteen sequence alterations were identified, and eight of them were novel including two novel nonsynonymous SNPs (L240V and I713V). Except the common I264V polymorphism, no other previously reported disease-causing or disease-susceptibility mutations were found. The novel I713V mutation was observed in three (3.7%) patients with HTG. One intronic SNP, IVS5+30C>T (rs10038177), showed significantly higher frequency of minor allele T in HTG patients (16.5%) than in controls (1.3%; Odds ratio [OR]=15.0, p=7.9×10^−7^, Bonferroni corrected p=1.5×10^−5^). Haplotype GTA, which is composed of rs13153937, rs10038177, and rs11241095, was significantly associated with HTG (OR=22.5, p=0.002, Bonferroni corrected p=0.013). Neither the individual SNPs nor haplotypes of *WDR36* were associated with NTG or JOAG (Bonferroni corrected p>0.05).

**Conclusions:**

Findings in this study suggest *WDR36* to be associated with sporadic HTG but not with NTG or JOAG. Our results also suggest a different mutation pattern of *WDR36* in the Chinese population from other ethnic populations.

## Introduction

Glaucoma is a group of diseases resulting in an irreversible degeneration of the optic nerve. It is one of the leading causes of blindness worldwide, estimated to affect more than 60 million people by 2010 [1]. Primary open-angle glaucoma (POAG) and exfoliation glaucoma (XFG) are the most common forms of glaucoma in Caucasian populations. But it is noted that in the Chinese population, XFG is rare [2] and primary angle-closure glaucoma (PACG) occurs at a higher frequency than POAG [3]. Genetic factors play an important role in the development of these disorders [4,5]. A recent advance in this field is that lysyl oxidase-like 1 (*LOXL1*) was found as a major gene associated with XFG, accounting for more than 90% of XFG cases in most populations [6-9]. However, the genetics of POAG appears to be more complex as genes conferring significant susceptibility have not yet been identified.

Fourteen chromosomal loci have been designated glaucoma 1, open angle, A (*GLC1A*) to *GLC1N* for POAG [4,10]. From these loci, two genes have been identified as causative factors for POAG. Mutations in the myocilin gene (*MYOC*) at *GLC1A* primarily cause high-tension glaucoma (HTG) [11,12], and the optineurin gene (*OPTN*) at *GLC1E* appears to contribute to normal-tension glaucoma (NTG) [13,14]. Recently, the WD repeat domain 36 gene (*WDR36*) at *GLC1G* was identified as a new gene for POAG [15]. Disease-causing mutations in *WDR36* were found in both HTG [15-18] and NTG patients [15,18,19]. However, some other studies did not find an association between *WDR36* and POAG [20,21].

Although *WDR36* has been evaluated for association with POAG in several studies [15-18], the contribution of *WDR36* to the occurrence of POAG is still controversial due to inconsistency in reported associations. Further evaluation of this gene in more populations is needed. The purpose of this study is to determine the distribution of *WDR36* sequence variants in a cohort of Chinese patients with POAG.

## Methods

### Patients and control subjects

Patients with POAG were recruited from the Eye Clinic of the Taipei Veterans General Hospital (Taipei, Taiwan). POAG was defined as meeting all of the following criteria: exclusion of secondary causes (e.g., trauma, uveitis, steroid-induced glaucoma, or exfoliation glaucoma), Shaffer grade III or IV open iridocorneal angle on gonioscopy, and characteristic optic disc damage or typical visual field loss by Humphrey automated perimeter with the Glaucoma Hemifield test. Intraocular pressure (IOP) was determined by applanation tonometry. Control subjects were recruited from people who attended the clinic for conditions of senile cataract, floaters, refractive errors, or itchy eyes. They were excluded from glaucoma using the same criteria of diagnosis as the POAG patients after going through the same procedure of ophthalmic examination. The project was approved by the Ethics Committee for Human Research at the Chinese University of Hong Kong. Informed consent was obtained from all study subjects after explaining the nature and possible consequences of the study in accordance with the tenets of the Declaration of Helsinki.

A cohort of 135 unrelated patients with POAG and 77 unrelated control subjects without glaucoma were included in this study. The demographic and clinical features of the study subjects are summarized in Table 1. The POAG group comprised 110 males and 25 females. Their age at diagnosis ranged from 16 to 85 years (mean±SD: 61±15.0 years). The highest IOP ranged from 13 to 77 mmHg (mean±SD: 24±8.0 mmHg). The vertical cup-disc ratio from 0.7 to 1.0 (mean±SD: 0.8±0.09) and visual field loss were compatible with glaucoma in two consecutive Humphrey testing. In this POAG group, 11 patients were juvenile-onset POAG (JOAG) whose age at diagnosis was less than 35 years (mean±SD: 26.5±6.3 years), the highest IOP ranged from 24 to 77 mmHg (mean±SD: 32.5±7.8 mmHg), 82 patients had late-onset HTG with the highest IOP being greater than or equal to 22 mmHg (mean±SD: 25.7±5.6 mmHg), and 42 patients had late-onset NTG with the highest IOP being less than 22 mmHg (mean±SD: 17.9±2.3 mmHg). The control group had 58 males and 19 females whose age at inclusion ranged from 52 to 86 years (mean±SD: 72±8.5 years), and their highest IOP ranged from 8 to 21 mmHg (mean±SD: 16±3.0 mmHg), their vertical cup-disc ratio from 0.2 to 0.5 (mean±SD: 0.4±0.07), their visual fields within normal range, and they had no family history of glaucoma.

**Table 1:** Demographic and clinical features of the study subjects.

**Group**	**N**	**Sex (M/F)**	**Age at diagnosis (years)**		**Highest IOP (mmHg)**		**Vertical cup-disc ratio**	
			**Range**	**Mean±SD**	**Range**	**Mean±SD**	**Range**	**Mean±SD**
POAG	135	110/25	16–85	61.0±15.0	13–77	24.0±8.0	0.70–1.00	0.80±0.09
HTG	82	67/15	35–83	63.0±11.3	22–45	25.7±5.6	0.70–1.00	0.81±0.10
NTG	42	33/9	38–85	66.7±10.1	13–21	17.9±2.3	0.70–0.95	0.84±0.07
JOAG	11	10/1	16–34	26.5±6.3	24–77	32.5±7.8	0.70–1.00	0.80±0.09
Controls	77	58/19	52–86	72.0±8.5	8–21	16.0±3.0	0.20–0.50	0.40±0.07

For controls, age at diagnosis refers to age at inclusion.

All the subjects were Han Chinese living in Taiwan. They were recruited from the same eye clinic and had a similar ethnic background. The cases and controls were matched for sex with 81.5% and 75.3% being males in POAG patients and controls, respectively (p=0.29). Because of the age dependence of POAG, only controls older than 50 years of age were included.

### Mutation screening

Genomic DNA was extracted from 200 µl of whole blood using a commercial kit (Qiamp Blood Kit; Qiagen, Hilden, Germany). Quantification of extracted DNA was performed using NanoDrop ND-1000 spectrophotometer (NanoDrop Technologies, Wilmington, DE). All 23 coding exons and splicing sites of *WDR36* were amplified by polymerase chain reaction (PCR) followed by DNA sequencing. Previously reported primers [22] were used to obtain the initial amplicons. Initial PCRs were performed on a thermal cycler (model 9700; Applied Biosystems [ABI], Foster City, CA) in a total volume of 25 µl containing 200 ng of genomic DNA, 0.4 µM of each primer, 200 mM dNTPs, 20 mM Tris-HCl (pH 8.0), 50 mM KCl, 1.5−3.0 mM MgCl_2_, and 1 U of Taq DNA polymerase (AmpliTaq Gold; ABI). Cycling conditions were as follows: first denaturation step of 12 min at 94 °C, 35 cycles of denaturation (94 °C for 40 s), annealing (primer-specific annealing temperature for 60 s), elongation (72 °C for 40 s), and a final single elongation step of 7 min. The PCR products were electrophoresed on 2% agarose gel and visualized using a video gel documentation system (Gel-Doc 2000; Bio-Rad Laboratories, Hercules, CA) to check for the quality. The PCR products were then purified with ExoI-SAP kit (USB Corp., Cleveland, OH) to remove unconsumed dNTPs and primers. A second PCR was performed using the sequencing primers as previously described [22] on a thermal cycler (model 9700; ABI) to incorporate the sequencing dyes (BigDye^®^ Terminator v3.1 cycle sequencing kit; ABI) using a protocol of 25 cycles of denaturation (96 °C for 10 s), annealing (50 °C for 5 s), and elongation (60 °C for 4 min). Sequence data were then aligned using Sequence Navigator analysis software (version 1.0.1; ABI) and compared with the published *WDR36* sequence (NM_139281). *MYOC* and *OPTN* were screened for sequence alterations by PCR and direct sequencing as previously described [14,23].

### Statistical analysis

Statistical analyses were performed using PLINK (version 1.04). PLINK is a free statistical analysis toolset, designed to perform a range of basic and large-scale analyses for genome-wide association studies in a computationally efficient manner [24]. Hardy–Weinberg equilibrium was assessed using an exact test [25]. The frequencies of the *WDR36* variants between patients with HTG, NTG, or JOAG and controls were compared using Fisher’s exact test. Linkage disequilibrium (LD) analysis was performed using Haploview (version 4.1) [26]. Haplotype frequencies were estimated using the standard E-M algorithm and tested using χ^2^ test. Omnibus p values were obtained from the omnibus tests. Specific p values were obtained from the haplotype-specific tests. The odds ratio (OR) and 95% confidence interval (CI) were calculated for each individual haplotype compared to all the other haplotypes. Multiple comparisons were corrected using the Bonferroni method. Despite the Bonferroni correction being considered conservative, especially for small samples, we used it to reduce the possibility of false-positive results and to report our significant association of *WDR36* with HTG with more confidence. Disease-causing mutations were defined (1) to alter the amino acid sequence of the corresponding protein and (2) to be completely absent from the control population or significantly more common in the POAG population [27].

## Results

Nineteen single nucleotide polymorphisms (SNPs) were identified, eight of which were novel (Table 2). All SNPs followed Hardy–Weinberg equilibrium in the control group (p>0.05). Three were nonsynonymous SNPs. Two were novel (L240V and I713V) while I264V had been previously reported [15]. The only likely disease-causing mutation, I713V, was found in three patients with HTG (3.7%) and was absent in patients with NTG or JOAG and controls (p=0.25). The L240V variant was found in one control subject (p=0.48). The previously reported common SNP, I264V (rs11241095), was evenly distributed between HTG, NTG, JOAG, and control groups (p>0.51).

**Table 2:** *WDR36* sequence variants observed in 135 POAG patients and 77 control subjects.

**Location**	**Sequence Change (major allele A> minor allele B)**	**Codon Change**	**SNP ID**	**Minor Allele (B) Frequency (%)**				**Genotype Frequency (BB/AB/AA)**			
				**HTG (n=164)**	**NTG (n=84)**	**JOAG (n=22)**	**Control (n=154)**	**HTG (n=82)**	**NTG (n=42)**	**JOAG (n=11)**	**Control (n=77)**
Nonsynonymous variant											
Exon 6	c.718C>G	L240V	Novel	0 (0.0)	0 (0.0)	0 (0.0)	1 (0.6)	0/0/82	0/0/42	0/0/11	0/1/76
Exon 7	c.790A>G	I264V	rs11241095	37 (22.6)	16 (19.0)	6 (27.3)	36 (23.4)	4/29/49	2/12/28	0/6/5	3/30/44
**Exon 18**	**c.2137A>G**	**I713V**	**Novel**	**3 (1.8)**	0 (0.0)	0 (0.0)	0 (0.0)	**0/3/79**	0/0/42	0/0/11	0/0/77
Synonymous variant											
Exon 4	c.540T>A	T180T	Novel	1 (0.6)	0 (0.0)	0 (0.0)	0 (0.0)	0/1/81	0/0/42	0/0/11	0/0/77
Exon 18	c.2142C>G	V714V	rs17624563	16 (9.8)	9 (10.7)	1 (4.5)	9 (5.8)	0/16/66	0/9/33	0/1/10	0/9/68
Exon 19	c.2181A>T	V727V	rs13186912	30 (18.3)	15 (17.9)	6 (27.3)	33 (21.4)	4/22/56	2/11/29	0/6/5	2/29/46
Intronic variant											
Intron 3	IVS3–113G>A	-	rs13153937	49 (29.9)	19 (22.6)	6 (27.3)	41 (26.6)	9/31/42	4/11/27	1/4/6	5/31/41
**Intron 5**	**IVS5+30C>T**	**-**	rs10038177	**27 (16.5)**	5 (6.0)	2 (9.1)	2 (1.3)	**6/15/61**	2/1/39	0/2/9	0/2/75
Intron 8	IVS8+38A>G	-	Novel	0 (0.0)	0 (0.0)	0 (0.0)	1 (0.6)	0/0/82	0/0/42	0/0/11	0/1/76
Intron 8	IVS8+45C>G	-	Novel	0 (0.0)	1 (1.2)	0 (0.0)	0 (0.0)	0/0/82	0/1/41	0/0/11	0/0/77
Intron 9	IVS9–81T>C	-	No SNP ID	6 (3.7)	4 (4.8)	1 (4.5)	9 (5.8)	0/6/76	0/4/38	0/1/10	1/7/69
Intron 13	IVS13+89G>A	-	rs34962120	31 (18.9)	19 (22.6)	5 (22.7)	28 (18.2)	4/23/55	5/9/28	0/5/6	3/22/52
Intron 14	IVS14+89C>A	-	rs13161853	62 (37.8)	23 (27.4)	7 (31.8)	42 (27.3)	15/32/35	2/19/21	1/5/5	9/24/44
Intron 16	IVS16–30A>G	-	rs17553936	40 (24.4)	17 (20.2)	3 (13.6)	33 (21.4)	7/26/49	2/13/27	0/3/8	3/27/47
Intron 17	IVS17+32T>G	-	Novel	0 (0.0)	1 (1.2)	0 (0.0)	0 (0.0)	0/0/82	0/1/41	0/0/11	0/0/77
Intron 17	IVS17+59A>C	-	Novel	0 (0.0)	0 (0.0)	0 (0.0)	1 (0.6)	0/0/82	0/0/42	0/0/11	0/1/76
Intron 18	IVS18+216C>T	-	rs17554123	16 (9.8)	9 (10.7)	1 (4.5)	14 (9.1)	0/16/66	0/9/33	0/1/10	0/14/63
Intron 18	IVS18–10T>A	-	Novel	1 (0.6)	0 (0.0)	0 (0.0)	0 (0.0)	0/1/81	0/0/42	0/0/11	0/0/77
Intron 21	IVS21+60G>C	-	rs2290680	19 (11.6)	9 (10.7)	1 (4.5)	9 (5.8)	0/19/63	0/9/33	0/1/10	0/9/68

One disease-causing mutation, I713V, was found in three patients with HTG (3.7%) and was absent in patients with NTG or JOAG and in controls. The minor allele T of IVS5+30C>T (rs10038177) was found in a significantly higher frequency in HTG patients than in controls (p=7.9×10^−7^, Bonferroni corrected p=1.5×10^−5^; OR=15.0, 95% CI: 3.50, 64.2). The allele T carriers (genotypes TT/CT) had an increased HTG risk (p=2.3×10^−5^; OR=12.9, 95% CI: 2.91, 57.2) compared to non-allele T carriers (genotype CC). None of the other SNPs of *WDR36* was found to be associated with HTG, NTG, or JOAG (p>0.05).

Three synonymous SNPs, one of which was novel (T180T), and 13 intronic SNPs were found, including five that had not been previously reported (Table 2). None of the intronic changes or synonymous SNPs was expected to affect splice sites. The minor allele T of IVS5+30C>T (rs10038177) was found in a significantly higher frequency in HTG patients than in controls (p=7.9×10^−7^, Bonferroni corrected p=1.5×10^−5^; OR=15.0, 95%CI: 3.50, 64.2). The allele T carriers (genotypes TT/CT) had an increased HTG risk (p=2.3×10^−5^; OR=12.9, 95% CI: 2.91, 57.2) compared to non-allele T carriers (genotype CC). None of the other SNPs of *WDR36* was found to be associated with HTG, NTG, or JOAG (p>0.05).

Haplotype GTA, composed of rs13153937, rs10038177, and rs11241095, was significantly associated with HTG (p=0.002, Bonferroni corrected p=0.013, OR=22.5; Table 3). No haplotypes of *WDR36* were associated with NTG or JOAG (Bonferroni corrected p>0.09, data not shown).

**Table 3:** Haplotype analysis of *WDR36* in 82 HTG patients and 77 control subjects.

**Haplotype**	**Haplotype Frequency (%)**		**p**	**OR (95% CI)**
	**HTG (n=164)**	**Controls (n=154)**		
Block 1: rs13153937, rs10038177, rs11241095. Omnibus p=0.0009				
GCA	57.7	67.3	0.07	0.66 (0.42, 1.05)
ACG	12.3	17.5	0.20	0.66 (0.35, 1.23)
ACA	8.1	8.0	0.98	1.02 (0.45, 2.28)
GCG	5.8	5.9	0.97	0.98 (0.38, 2.50)
**GTA**	**6.5**	**0.1**	**0.002**	**22.5 (1.31, 386)**
ATA	5.5	1.2	0.03	4.41 (0.94, 20.8)
ATG	4.0	0.0	0.01	13.9 (0.78, 247)
Block 2: rs34962120, rs13161853. Omnibus p=0.01				
GC	60.3	64.2	0.47	0.85 (0.54, 1.33)
GA	20.8	17.6	0.47	1.23 (0.70, 2.16)
AA	17.0	9.6	0.05	1.91 (0.98, 3.73)
AC	1.9	8.6	0.008	0.20 (0.06, 0.72)
Block 3: rs17553936, rs17624563, rs17554123, rs13186912, rs2290680. Omnibus p=0.0008				
ACCAG	48.3	63.8	0.007	0.53 (0.34, 0.83)
GCCAG	15.9	13.4	0.55	1.19 (0.64, 2.22)
ACCTG	12.5	13.1	0.87	0.96 (0.50, 1.85)
AGCAG	6.7	0.6	0.006	11.0 (1.40, 86.3)
GCCTG	1.7	6.0	0.05	0.30 (0.08, 1.13)
GCTAG	5.5	1.2	0.04	4.41 (0.94, 20.8)
ACCTC	4.6	0.6	0.03	7.33 (0.90, 59.8)
ACTAG	1.7	1.2	0.70	1.42 (0.23, 8.59)
AGCAC	3.0	0.2	0.07	10.7 (0.58, 194)

Only haplotypes with overall frequency above 1% were shown. Haplotype GTA was significantly associated with HTG (p=0.002, Bonferroni corrected p=0.013; OR=22.5). The other haplotypes were not significant after Bonferroni correction (Bonferroni corrected p>0.05).

## Discussion

In the present study, three patients with HTG (3.7%) carried a novel disease-causing mutation (I713V). They were free of disease-causing mutations in *MYOC* and *OPTN* [28]. The absence of this mutation in 154 human normal control chromosomes suggests that it might affect the normal function of the WDR36 protein. This mutation is located within a domain of WDR36 named mini-chromosome maintenance (MCM) protein 5 (amino acids 703–718 and 873–885). MCM proteins are a family of eukaryotic replication factors required for the initiation of DNA replication. MCM5 directly interacts with the Stat1 protein (signal transducer and activator of transcription) to enhance Stat1-mediated transcription activation [29]. These findings suggest that WDR36 might be involved in transcription activation. However, the replacement of an isoleucine by a valine would only mildly alter the hydrophobicity in this region. The conformational structure of the protein is unlikely to be disrupted. It is also possible that the I713V mutation is a benign polymorphism that does not affect the function of WDR36. Further functional studies are required to elucidate the exact role of this novel mutation.

In the present study, we did not find the previously reported disease-causing mutations (N355S, A449T, R529Q, and D658G) and disease-susceptibility mutations (L25P, A163V, and Y216P) [15]. Except the common I264V polymorphism, we also did not identify other reported nonsynonymous SNPs (P31T, D33E, Y97C, D126N, H212P, M283R, A353S, D354N, I361V, T403A, H411Y, H411L, C470Y, P487R, I604V, S664L, M671V, and N668H), which were identified from different populations [15-20]. As a whole, our results suggest a different mutation pattern for *WDR36* in the Chinese population from other ethnic populations. The common I264V variant was reported to have a significantly higher frequency in Japanese patients with HTG than controls [17]. However, we found similar frequencies of this variant in our POAG patients and controls, which are consistent with two other studies of Caucasian populations [16,18], arguing that I264V may be a benign polymorphism.

The total mutation prevalence of *WDR36* in Chinese patients with HTG (3.7%) is similar to those in the original study of a Caucasian population from USA (3.2%) [15] and one report from Germany (3.7%) [18]. However, it is lower than that in another study of the USA Caucasian population (17%) [16], and higher than the prevalence found in one report of the Japanese population (0.7%) [17]. It is noteworthy that neither individual SNPs nor haplotypes in *WDR36* were associated with NTG in our study while *WDR36* mutations had been identified in both HTG [15,16,18] and NTG patients [15,18,19] in Caucasian populations. Intriguingly, the similar results of negative association of *WDR36* with NTG were also reported in a Japanese population [17], suggesting that the variants of *WDR36* may affect only HTG in Asian populations.

In the present study, the association analysis was used for the first time to explore ancestral alleles in *WDR36* that may pose risk to glaucoma. We found that one intronic SNP of *WDR36* (IVS5+30C>T) was significantly associated with HTG (p=7.9×10^−7^). But we did not find significant association of this variant with the highest recorded IOP and vertical cup-disc ratio (p>0.2; data not shown). Conflicting association of SNP IVS5+30C>T with glaucoma has been reported. Analysis of the published genotype data from a study of a USA population revealed that this SNP was significantly associated with HTG and JOAG (p=1.8×10^−9^) [16]. However, another study of a German population did not demonstrate significant association of this SNP with POAG (p=0.27) [18]. Our recent study of a northern Chinese population also did not identify significant association of this SNP with HTG and JOAG (p=0.22) [30]. Unfortunately, we could not directly compare these data with our results as the HTG patients were not separately analyzed in those studies [16,18,30]. Notably, we did not find functional mutations in *WDR36* in linkage disequilibrium with this common intronic SNP. The only potential disease-causing mutation (I713V) identified in the present study is a rare mutation, which is clearly not the source of this significant association. It is possible that the true disease-causing mutations might be located in the promoter or introns of *WDR36* that were unable to be detected in this study. Another possibility is that SNP IVS5+30C>T might be in linkage disequilibrium with disease-causing mutations in a neighboring gene. This latter proposition has some indirect supportive evidence. Two studies reported *WDR36* not associated with POAG in several families, which were linked to *GLC1G* [15,31]. Also, no association was found between *WDR36* and unrelated patients with POAG in some populations [20,21]. Although the sample size was not large in our study, it did not compromise the significant association of SNP IVS5+30C>T with HTG. However, other POAG associated sequence variants could still be missed. A large-scale study is warranted.

The function of WDR36 was recently determined using zebrafish models [32]. It is the functional homolog of yeast U3 small nucleolar RNA-associated protein 21 (Utp21), which is cell essential and functions in the nucleolar processing of 18S rRNA [32]. In yeast models, certain *Utp21* variants homologous to glaucoma-associated variants in human *WDR36* cause functional defects in a stress-induced-phosphoprotein 1 (*Sti1*) mutant background, arguing that *WDR36* contributes to polygenic forms of glaucoma [33]. Loss of Wdr36 function leads to an activation of the p53 stress response pathway while p53 may act as a transcriptional activator [32]. Collectively, WDR36 might be involved in transcription activation either through its MCM5 domain or through the p53 stress response pathway [29,32]. Although genetic studies of *p53* variants have shown inconsistent association with POAG [34-37], it has been suggested that co-inheritance of defects in p53 pathway genes may influence the impact of *WDR36* variants on POAG [32]. *WDR36* may affect the disease severity of patients with POAG that is caused by mutations in *MYOC* [16]. We found that *OPTN* may interact with *MYOC *and contribute to the development of POAG [38]. This finding was further supported by an in vitro study of human trabecular meshwork cells that *OPTN* overexpression induced an upregulation of *MYOC* [39]. Although WDR36 has been shown to function in 18S rRNA processing and transcription activation [32], the exact role of WDR36 in glaucoma remains unclear. It has been suggested that WDR36 may participate in T-cell activation [40]. T-cell responses may be involved in optic nerve degeneration in glaucoma [41]. These findings indicate that WDR36 may contribute to glaucoma by modifying optic nerve degeneration. However, further studies are needed to address how WDR36 may influence POAG.

In summary, our data suggests the association of *WDR36* with sporadic HTG but not with NTG or JOAG. Our results also suggest a different mutation pattern of *WDR36* in the Chinese population from other ethnic populations.
